# Recipe for IBD: can we use food to control inflammatory bowel disease?

**DOI:** 10.1007/s00281-017-0658-5

**Published:** 2017-11-09

**Authors:** Mario Witkowski, Marco Witkowski, Nicola Gagliani, Samuel Huber

**Affiliations:** 1 Institute of Medical Microbiology and Hygiene, University of Mainz Medical Centre, Mainz, Germany; 2 Department of Internal Medicine and Cardiology, Campus Benjamin Franklin, Charité - Universitätsmedizin, Berlin, Germany; 3 Department of Medicine, University Medical Center Hamburg-Eppendorf, Hamburg, Germany; 4 Department of General, Visceral and Thoracic Surgery, University Medical Center Hamburg-Eppendorf, Hamburg, Germany; 5 Immunology and Allergy Unit, Department of Medicine, Solna, Karolinska Institute, 17176 , Stockholm, Sweden

## Abstract

The mucosal immune system and the microbiota in the intestinal tract have recently been shown to play a key role in the pathogenesis of inflammatory bowel disease (IBD). Both of these can be influenced by food. Thus, we propose dietary intervention as a therapeutic option for IBD. In this review, we discuss the interaction of the intestinal mucosal immune system and the intestinal microbiota in the context of IBD. In addition, we discuss the impact of food components on immune responses in IBD. Finally, we address the current evidence of how this interaction (i.e., immune system–microbiota) can be modulated by food components, pre/probiotics, and fecal microbiota transplantation (FMT) and how these approaches can support intestinal homeostasis. By gathering the vast amount of literature available on the impact of food on IBD, we aim to distinguish between scientifically sound data and theories, which have not been included in this review.

## The role of the immune system and the intestinal microbiota in IBD

IBD is a group of chronic disorders characterized by relapsing inflammation of the gastrointestinal (GI) tract. The most common entities are Crohn’s Disease (CD) and Ulcerative Colitis (UC). While CD is characterized by a segmental transmural inflammation and granulomatous lesions of the whole GI tract, inflammation in UC is mostly restricted to the colon and the rectum. Both incidence and prevalence of IBD are currently increasing worldwide [1].

Although the etiology of IBD has not yet been fully elucidated, more than a hundred genetic risk loci have been identified so far. These genetic studies point toward an important role of the immune system and its interaction with the microbiota in the development of IBD. The majority of these loci are indeed linked to loss of barrier function (e.g., GNA12), to host interaction with bacteria (e.g., NOD2), or to immune pathways (e.g., IL-23) implicated in the control of cytokines, such as interleukin (IL)-17 and IL-22 [2]. Notably, these cytokines are profoundly involved in the regulation of the intestinal mucosal barrier homeostasis and are triggered by signals from the intestinal microbiota [3–5]. This reinforces the idea that a deregulated immune response to intestinal microbiota is involved in IBD pathogenesis [6].

In this section, we will refresh the knowledge on the intestinal microbiota, how it interacts with the immune system, and furthermore the role of the key cytokines (IL-23, IL-17, IL-22, IL-10), which result from this interaction in the context of IBD.

### Intestinal microbiota

The human body coexists with around 10^13^ bacterial organisms, which colonize different sides of the body, such as the intestine and the skin [7]. In the intestine, we refer to these bacteria as the intestinal microbiota. The dominant phyla are the Firmicutes (64%) that mostly comprise bacteria of the Clostridia class, followed by Bacteroidetes, which account for approximately 23% of the intestinal flora, Proteobacteria, and Actinobacteria [8, 9]. The physiological metabolic activity of the microbiota is beneficial for the host. The intestinal microbiota promotes, for example, the fermentation of indigestible carbohydrates to, e.g., short-chain fatty acids (SCFA) and absorption of complex lipids or vitamin synthesis [10–12].

An altered microbiota has been associated with IBD, and a variety of extra-intestinal diseases, such as obesity, type 1 and type 2 diabetes, asthma, and liver disease highlighting the systemic impact of the intestinal flora [13, 14]. Sequencing analysis of intestinal biopsies or fecal samples of IBD patients showed a different composition of mucosa-associated and fecal microbiota compared to healthy controls with a reduced microbial biodiversity as a hallmark of this disease [15]. More detailed analysis revealed that certain bacteria species protect from IBD while others appear to be pathogenic.

Various studies demonstrated a marked reduction of Firmicutes within the microbiota of patients with IBD [16–19]. Within the Firmicutes-phylum, in particular, the species *Faecalibacterium prausnitzii* was shown to be protective in chemically induced colitis in mice. This observation has been further sustained by human twin studies [20, 21].

Bacterial species from the Proteobacteria phylum have mainly been reported as holding pathogenic potential in the development of IBD. For example, *Escherichia coli* (*E. coli*) was shown to be more abundant in patients with active IBD [22]. In particular, adhesive-invasive E. coli (AIEC) is enriched in the inflamed ileal mucosa of IBD patients and might trigger local inflammation [23, 24].

Moreover, sulfate-reducing bacteria (SRB), most of which belong to the Deltaproteobacteria class (Proteobacteria phylum), are increased in IBD and have been shown to promote a chemically induced colitis in mice underlining a possible role in IBD pathogenesis [25, 26]. However, also species from other bacterial phyla, such as Bacteroidetes, have shown pathogenic properties. For example, in a mouse model that is susceptible for colitis, the fecal microbiota was characterized by expansion of the *Prevotella* genus and the TM7 phylum [27].

### Interaction between the immune system and the microbiota

The immune system has several sensors, which detect the microbiota and its changes and consequently can promote intestinal inflammation or maintain homeostasis. For the maintenance of a physiological interaction, a highly regulated network of microbial sensor systems has evolved.

The mucosal immune system recognizes the conserved microbe-associated molecular patterns (MAMP) via pattern recognition receptors (PRR), such as Toll-like receptors (TLR) and nucleotide oligomerization domain (NOD)-like receptors (NLR). These specialized receptors respond to a variety of triggers, including unmethylated double-stranded DNA, single-stranded RNA, and bacterial products [28]. Ligand-receptor interaction induces the recruitment of signaling adaptors, such as myeloid differentiation primary response gene 88 (MyD88) and TIR domain-containing adapter-inducing interferon-β (TRIF). Toll-like receptors are expressed by various cells of the immune system including dendritic cells (DCs), macrophages, T cells and B cells, and also by epithelial cells [29].

A variety of bacterial products have been reported as TLR ligands, such as lipopolysaccharide (LPS) from gram-negative bacteria, peptidoglycans from gram-positive bacteria, or flagellin derived from flagellated bacterial species [30]. Sensing of these bacterial components by individual TLRs triggers pro-inflammatory cytokines, such as TNF-α and can induce also type I interferons [31]. The NLR receptor family features a wide range of functions. Besides bacterial products, NLRs also sense cell stress signals, for example, via reactive oxygen species (ROS) or via oxidized mitochondrial DNA [32, 33]. Downstream signals of NLR activation have also been shown to induce production of anti-microbial peptides as well as goblet cell mucus production thereby directly promoting mucosal barrier defense [34, 35].

Bacterial products are also sensed by inflammasomes, which are multiprotein complexes assembling with the adapter protein apoptosis-associated speck-like protein (ASC) and procaspase-1. In response to PAMPs, activated caspase-1 cleaves the pro-forms of the pro-inflammatory cytokines IL-1β and IL-18 [28]. Impaired microbial sensing has been shown to impact the microbial composition and to promote inflammation: global deletion of inflammasome components, such as ASC or NLRP6, a member of the NLR family, associates with dysbiosis with microbial shifts, and a subsequent colitis [27].

### Resulting cytokine: IL-23 mediates the interaction between the microbiota and the immune system

Intestinal immune responses and intestinal tissue homeostasis are regulated by cytokines, which are produced when the abovementioned sensory systems are engaged. Within these cytokines IL-23 has a prominent role in the intestine, specifically in IBD as highlighted by genome-wide association studies (GWAS) [2, 36]. Interleukin -23 is an important downstream signal of TLR-activation, and IL-23 signaling has been shown in mouse and human studies to promote IBD [37–39]. Important triggers of IL-23 production are bacterial products, such as flagellin. Flagellin is produced by bacteria belonging to the clostridia class of the Firmicutes phylum, but also by members of the Proteobacteria phylum, such as *Escherichia coli*. Interestingly, patients with CD show flagellin-specific circulating antibodies in contrast to healthy controls underlining the immunogenicity of flagellin in the context of intestinal inflammation [40]*.* One of the cellular sources of IL-23 are dendritic cells located in the lamina propria of the epithelial layer, which produce IL-23 upon sensing bacterial-derived flagellin via TLR-5 [41]. Recently, also CXCR1+ CXCR2+ neutrophils located in the inflamed colonic mucosa of patients with IBD have been identified as a cellular source of IL-23 [42]. IL-23 promotes an immune response characterized by the induction of other two key cytokines IL-17 and IL-22. These cytokines are secreted by both innate and adaptive immune cells, and they will be further discussed in the following paragraphs.

### Resulting cytokine: IL-17 and its protective and pathogenic properties

In particular, the role of IL-17 in IBD became the subject of controversial debate. Among the IL-17 family members, the biological role of IL-17A and IL-17F are best characterized. On the one hand, in line with the GWAS, IL-23-dependent secretion of IL-17 was shown to promote T cell-mediated colitis [43, 44]. Recently, also IL-17A derived from group 3 innate lymphoid cells (ILC) in response to IL-23 has been shown to mediate intestinal inflammation in an innate colitis model [45]. Likewise, IL-17F has been reported to play a pathogenic role in colitis development with either redundant or different functions compared to IL-17A [43, 46]. On the other hand, there are studies reporting a protective effect of IL-17A in various colitis models, such as dextran sulfate sodium (DSS)-induced or T cell-mediated colitis models [47, 48]. Insights in mechanisms underlying the protective capacity of IL-17A have recently been delineated: during DSS-colitis IL-17A-dependent regulation of tight junctions can limit excessive intestinal permeability and therefore promote barrier integrity. Of note, in this model, IL-17A was produced by γδ T cells in an IL-23-independent manner [49]. Beside ILC, also T_H_17 cells can produce IL-17. These cells are a subtype of effector T helper cells, which express high levels of IL-17A and IL-17F and which are highly enriched in the inflamed mucosal tissue of IBD patients [50, 51]. Interestingly, some commensal bacteria, such as segmented filamentous bacteria (SFB), can induce T_H_17 cells [52].

In conclusion, these findings suggest that IL-17A can have dual functions, which might depend on the cellular source, on the environment, such as the microbiota, and on the type of intestinal pathology. Of note, IL-17A blockade using an antibody was not effective in IBD in humans [53]. However, by understanding the context-dependent effect of IL-17A, one may be able to identify a subset of IBD patients, which might benefit from this therapy.

### Resulting cytokine: IL-22 and its dual role at mucosal surfaces

IL-22 acts also downstream of IL-23 and plays a central role in promoting barrier homeostasis by regulating genes encoding molecules associated with tissue repair or antimicrobial properties [54]. Both adaptive immune cells and cells of the innate immune system can produce IL-22, and early studies demonstrated that adaptive and innate IL-22 protects from IBD [55]. In particular, IL-22 produced by innate lymphoid cells is essential for intestinal barrier control in a murine model for enteropathogenic *E. coli* infection [56]. Innate -derived IL-22 is also crucial in controlling SFB [57].

However, as shown for the IL-17 family, IL-22 can also mediate pathogenic activity in intestinal inflammation. For example, T cell-derived IL-22 promotes colitis upon transfer of memory cells into Rag1-deficient recipients [58]. Furthermore, IL-22-mediated antimicrobial response suppresses the commensal flora and indirectly favors the colonization of enteropathogenic bacteria, such as Salmonella enterica [59].

In conclusion, IL-22 has pathogenic and protective properties. Therefore, a tight control is essential. This control is exerted on at least two levels. First, as mentioned above, the production of IL-22 is regulated by IL-23. Second, there is an endogenous inhibitor of IL-22, namely IL-22 binding protein (IL-22BP). IL-22BP binds and blocks IL-22 activity. IL-22BP can be produced by DCs, granulocytes, and T cells [60–62]. Interestingly, T cell-derived IL-22BP was shown to be upregulated in IBD and to promote IBD via blockade of the beneficial effects of IL-22 [62].

### Resulting cytokines: IL-10 and immune tolerance

Intestinal homeostasis is the result of a fine-tuned balance of factors promoting immune responses and signals mediating immune tolerance. A key player in limiting immune responses is the anti-inflammatory cytokine IL-10 [63]. Polymorphisms leading to a loss of function of the IL-10 receptor or IL-10 itself are associated with early-onset IBD reinforcing the theory of a pathogenic loss of immune tolerance to intestinal microbes [64, 65]. Microbial products can drive IL-10 expression by macrophages and DCs. By various mechanisms, such as limiting antigen presentation and the expression of costimulatory molecules or by down regulation of pro-inflammatory cytokines, IL-10 mediates immune tolerance [66, 67]. For example, germ-free mice colonized with the human commensal species *Bacteroides fragilis* show induction of IL-10-producing regulatory T (Treg) cells via lipopolysaccharide A (LPA) [68]. In line with these results, administration of LPA itself has been shown to be protective in different experimental models of colitis [68, 69]. Various clostridia species have also been reported to induce IL-10-producing Treg cells, and oral inoculation of these bacterial strains mediates amelioration of chemically induced colitis underlining the protective role of IL-10 in these models [70, 71]. Also, signaling of the NLR family member NOD2 induces IL-10 expression, and NOD2 mutations are associated with Crohn’s disease [72, 73].

Overall, IL-10 is a crucial player in controlling inflammatory responses in the intestine. In particular, understanding the microbiota-derived signals promoting Treg cell responses will help to optimize flora-based therapeutic approaches for IBD.

## Impact of food components on the intestinal microbiota and on the local immune response in IBD

Epidemiological studies support the role of environmental factors in pathogenesis of IBD. In particular, the association between dietary intake and incidence of IBD points to food components as a potential pathogenic agent. Indeed, certain food components, such as polyunsaturated fatty acids (PUFA), omega-6 fatty acids, and meat seem to predispose for IBD, whereas fibers and fruits or vegetables were associated with a decreased risk of CD or CU, respectively [74].

**Fig. 1:**
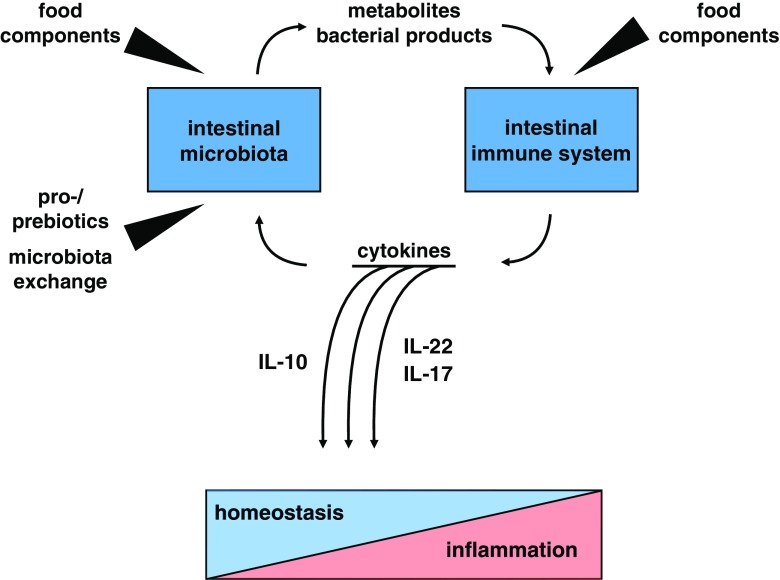
Schematic overview of interactions between diet, microbiota and immune system in the intestine

While there is little evidence of food components having a direct impact on IBD, they do impact in an indirect fashion (Fig. 1). Food components essentially influence the intestinal homeostasis by modulation of the microbiota and subsequent activation of the immune system. Indeed, a controlled-feeding study of ten donors showed that the microbiota responds by compositional change within 24 h of initiating a low fat/high fiber or high fat/low fiber diet [75]. Wu *et al.* showed that different diets are associated with specific bacterial clusters of the microbiota. Diets based on protein and animal fat compared to carbohydrates were associated with two enterotypes according to the levels of *Bacteroides* and *Prevotella*, both of which are genera, which are involved in colitis development [75]. These observations are also supported by animal studies showing that a high fat diet was associated with an increase in Firmicutes and a reduction of species from the Bacteroides phylum [76]. Of note, high-fat diets were also shown to promote chemically induced colitis in mice and were associated with expression of inflammatory cytokines and a decreased frequency of regulatory T cells [77]. On the other hand, high-fat diet was also reported to decrease intestinal T_H_17 cells [78]. Collectively, these data indicate that basic dietary components, such as fat and carbohydrates, can specifically modulate the microbial composition of the intestine.

Further, important modulators of the microbiota are dietary fibers. A diet low in fibers (considered the Western diet), results in a loss of the microbial diversity as observed in IBD patients [79]. Fermentable fiber is metabolized by the intestinal microbiota, mainly by clostridia species, to SCFA, such as acetate and butyrate. SCFA are strong modulators of the intestinal immune system, and their effects are coordinated by specific metabolic programs of immune cells [80].

The intestinal microbiota is also involved in the synthesis and the metabolism of various vitamins, such as B-group vitamins and vitamin K. On the other hand, vitamins have been shown to modulate the microbiota, and to act directly on the immune system; vitamin A and its down-stream metabolite retinoic acid (RA) are involved in multiple immune processes, such as Treg homeostasis and IgA antibody production [81, 82].

Deciphering the role of food components in modulating the intestinal immune system might help to use this potential to expand treatment options for IBD. In this section, we summarize current evidence of how certain food components (carbohydrates, dietary fiber, fat, and vitamins) modulate the intestinal immune system.

### Carbohydrates, dietary fiber, and fat

Several studies showed a positive, but still not consistent, association between high carbohydrate intake and an increased risk of IBD [74]. Based on sequencing analysis, carbohydrate consumption significantly correlates with abundance of the Firmicutes phylum and is associated with the *Prevotella*-enterotype [75]. Currently, there is a lack of studies investigating the specific effect of carbohydrates or withdrawal of them from diet in IBD. Thus, the exact role of carbohydrates in IBD is currently not clear.

However, there are several studies pointing toward a role of fat intake in IBD, although this role is still controversial. A positive association has been reported between high intake of saturated fats, mono- and polyunsaturated fatty acids (PUFA), and an increased risk of CD, whereas high total fats correlated with an increased CU risk [74]. Likewise, high-fat diets promote chemically induced colitis in mice and are associated with a decrease in the frequency of regulatory T cells [77]. Furthermore, milk-derived saturated fat can increase the availability of organic sulfur by pathological conjugation of hepatic bile acids, thereby promoting sulfate-reducing bacteria and an associated colitis in a mouse model [83].

In contrast to the pathogenic properties, beneficial effects of fat have also been reported. In particular, omega-3 (3-n) PUFA might play a protective role in IBD [84]. Indeed, during chronic colitis, transgenic mice synthesizing (n-3) PUFA de novo show reduced polarization of T_H_17 cells. A possible mechanism, by which PUFA control T_H_17 cell pathogenicity is the modulation of cholesterol-derived ligands for RORγt, the major transcription factor of T_H_17 cells and group 3 ILC [85].

Finally, dietary fiber has been linked to IBD. In a meta-analysis, high intake of dietary fiber was associated with a decreased risk of IBD [74]. As mentioned above, the metabolism of fiber requires the intestinal microbiota. Fermentable fiber is metabolized mainly by clostridia species, to SCFA, such as acetate and butyrate, which seem to exert the protective properties in IBD. Interestingly, fecal SCFA levels were shown to be decreased in IBD patients [86]. Furthermore, a protective role of SCFA is supported by animal studies. Indeed, orally administered SCFA protected mice from T cell-mediated colitis or intestinal lesions induced by cytostatic agents [87, 88]. Several potential mechanisms explain these protective properties. First, SCFA show direct anti-inflammatory activity. SCFA can, for example, suppress NF-κB and trigger expression of the Vitamin D receptor (VDR, see below) [89, 90]. Furthermore, SCFA were shown to modulate oxidative stress and to promote barrier function by enhancing tight junction assembly [91]. Of note, the beneficial effects of fiber on intestinal inflammation also involve activation of the NLRP3 inflammasome by hyperpolarization of the epithelial membrane [92].

Second, SCFA have indirect protective properties, which are mediated via regulatory CD4^+^ T cells. Indeed, butyrate and propionate were shown to promote extra-thymic Treg generation [93]. Furthermore, interaction of SCFA and G-protein-coupled receptors (GPRs), such as GPR43, has been shown to mediate enhancement of frequency and function of Tregs [88]. Besides this interaction with GPR, also the distinct metabolic programs of immune cells explain the effect of SCFA on regulatory CD4^+^ T cell generation. Compared to effector T cells, such as T_H_17 cells, which are reliant on glucose metabolism, the generation of regulatory CD4^+^ T cells has been shown to be selectively dependent on lipid oxygenation [80]. The increased expression of genes regulating fatty acid oxidation enables Tregs to effectively use SCFA as an energy source [94]. Of note, the protective effect of colonization by Clostridia from clusters IV and XIVa with induction of regulatory T cells might also be mediated by SCFA but underlying mechanisms still remain to be revealed [95].

In conclusion, several studies underline the central role of metabolites in controlling the balance of inflammatory and regulatory immune responses based on immune cell-specific metabolic programs.

### Vitamins D and A

Vitamin deficiency is a common feature of IBD. Malnutrition due to decreased food intake, increased requirements, malabsorption, and maldigestion as possible causes are being discussed. A recent retrospective study in IBD patients showed high frequencies of Vitamins D and A deficiency [96]. There is now growing evidence for immunological action of these vitamins, for both adaptive and innate immunity.

Indeed, several studies demonstrated that vitamin D enhances the development of T_H_2 cells, a T helper cell subset characterized by the production of the anti-inflammatory cytokines IL-4, IL-5, and IL-13. Furthermore, the suppression of T_H_17 cells and the induction of IL-10-producing Treg cells in response to vitamin D have been reported [97, 98]. Interestingly, TLR triggering has been shown to promote Vitamin D receptor expression and downstream signals including the production of anti-microbial peptides [99].

Furthermore, Vitamin D might influence IBD via interactions with the microbiota. Indeed, absence of the Vitamin D receptor can directly impact the intestinal microbiota with an increase in *Bacteroides*, which was associated with an increased susceptibility toward DSS-induced colitis [90].

In line with the mouse studies, there are also human data supporting the role of Vitamin D in IBD. First, VDR gene polymorphisms are linked to increased IBD susceptibility [90]. Second, in a prospective study, low Vitamin D levels were associated with increased risk of surgery and hospitalization of IBD patients, and oral Vitamin D supplementation was associated with a reduced risk of relapse in Chrohn’s disease (CD) in a randomized double-blind placebo-controlled study [100, 101]. In line with these results, a recent cross-sectional study also showed a positive correlation of Vitamin D deficiency and IBD disease activity [102].

Vitamin A is a group of nutritional organic compounds. The exact role of Vitamin A in IBD is currently unknown. However, there are some data linking vitamin A-derived metabolites to the immune response in the intestine. One example is retinol, which is one principal compound of the vitamin A group that is metabolized to retinoic acid (RA) by the sequential action of retinol dehydrogenase (RDH) and retinaldehyde dehydrogenase (RALDH) enzymes [103]. Dendritic cells are the major source of RA in the intestine that is produced in response to inflammatory signals. Local RA induces the conversion of naïve CD4^+^ T cells into Treg cells and inhibits the differentiation of T_H_17 cells thereby promoting immune tolerance [82, 104]. Furthermore, in a recent study, RA has been shown to induce IL-22 secretion by γδ T cells and innate lymphoid cells with protective effects in murine colitis models. By directly targeting the IL-22 promotor, RA enhanced production of antimicrobial peptides [105]. Thus, RA might impact IBD via IL-17 and IL-22-mediated effects.

## Therapeutic opportunities: pro/prebiotics, diet intervention, and fecal microbiota therapy

To maintain homeostasis, a fine-tuned balance between immune tolerance and immune activation toward the intestinal microbiota is critical. Regarding IBD patients, observations have shown that this critical balance is deregulated favoring chronic immune activation. There are several possible ways to reset this balance with the aim of treating IBD patients. The first is to target the microbiota directly by microbiota transplantation; the second is to target the microbiota indirectly by diet (Fig. 1). In addition, there are also several therapies, which aim to modify the microbiota composition, such as the use of probiotic bacteria, and different diets have already been tested in IBD.

This section will provide a summary of the most recent evidence relating to the role of probiotics, prebiotics, diets, and fecal microbiota transplantation in the treatment of IBD.

### Probiotics

An option to influence the composition of intestinal microbiota is the use of probiotics. Specific microorganisms are deemed probiotic based on their scientifically proven effect on the promotion of health or prevention and treatment of a specific disease. Probiotics, such as *Escherichia coli* Nissle 1917 and VSL#3 seem to be efficient in UC as outlined below. However, there is not sufficient evidence to support the use of probiotics in daily clinical practice in Crohn’s disease [106].

Patients with CU in remission treated with *Escherichia coli* Nissle 1917 compared to immunosuppressive therapy with 5-aminosalicylic acid showed a comparable clinical relapse rate [107]. Likewise, this probiotic shows the same efficacy in maintaining remission compared to 5-aminosalicylic acid [108]. Furthermore, VSL#3, which contains four strains of *Lactobacillus* was shown to induce a significantly higher rate of remission compared to placebo in mild-to-moderately active UC [109].

Probiotics show signs of exerting their effects via at least two mechanisms. First, probiotics are able to enhance mucosal barrier function. They were shown to promote secretion of IgA and mucins [110] and tight junction function [111, 112]. Second, probiotics interact with the local immune system. An example is that some probiotic strains can enhance regulatory T cell responses, downregulate the expression of pro-inflammatory cytokines, such as TNF-α and IFN-γ [113, 114], and induce the production of a variety of anti-inflammatory cytokines, e.g., IL-10 and TGF-β [115].

Finally, probiotics impact the IL-22-mediated antimicrobial response. It has been shown that tryptophan is metabolized by a subset of commensal *Lactobacilli*, a bacterial genus contained in many probiotic products, to indole-3-aldehyde (IAld). IAld induced AhR-dependent IL-22 production by ILC and mediated both antifungal activity and amelioration of chemically induced colitis [116].

### Prebiotics

Prebiotics are indigestible substances, mostly carbohydrates that can be metabolized by beneficial bacterial strains hence enhancing their activity. Prebiotics in clinical use include fructo-oligosaccharides (FOSs), inulin, galacto-oligosaccharides (GOSs), and soybean oligosaccharides that stimulate mainly bifidobacteria species [117]. There is not much clinical evidence of therapeutic efficacy of prebiotics in IBD.

Patients with active CD were randomized and treated with fructo-oligosaccharides (FOSs) versus placebo with no significant difference in clinical response [118]. In a more recent study, De Preter *et al.* observed a significantly reduced clinical disease activity after 4 weeks of treatment with a prebiotic combination of oligofructose and inulin [119]. In this trial, an increase of fecal butyrate was shown after prebiotic treatment. This SCFA has been shown to induce T cell apoptosis and suppression of IFN-γ-mediated inflammation in colonic epithelial cells [120]. Therefore, the observed impact of prebiotics is probably linked to promotion of probiotic effects including bifidobacteria-mediated increase in IL-10 expression [121].

### Diets: elemental and polymeric

Exclusive enteral nutrition (EEN) is a dietary therapy comprised of a period of 6–8 weeks of exclusive liquid feeding with either elemental or polymeric formulae. Elemental diets contain all essential nutrients and protein in the form of free amino acids whereas polymeric formulae comprise non-hydrolyzed proteins and long-chain triglycerides. These formulae were developed to improve nutrient absorption and to limit mucosal antigen exposure.

Interestingly, several studies demonstrated effectiveness of EEN in the treatment of CD equivalent to the use of corticosteroids mainly in pediatric patients [122, 123]. In contrast, results of trial testing the efficacy in treatment of adults, were apparently controversial [124, 125].

One possible reason for the discrepancy between the efficacy of ENT in pediatric and adult patients might be the higher chance of non-compliance by adult patients, which in turn might be related to an increased intolerance of the formula observed in adult patients.

The exact mechanism of EEN in IBD is unknown, but several possibilities are being explored. First, the effects might be linked to the intestinal microbiota. During the dietary treatment, profound modification of all predominant bacterial groups were observed with a decrease in microbiota diversity [126]. Interestingly, a reduction of the protective commensal *Faecalibacterium prausnitzii* was reported, which might help to explain a therapy failure in some cases [127]. Second, results from in vitro experiments showed that the components of EEN reduced inflammatory responses, which is supported by a human study showing reduced mucosal expression of IL-1β and IFN-γ after EEN treatment [128–130]. Finally, also promotion of barrier function has been reported: EEN based on a polymeric formula prevented TNF-α-mediated tight junction dysfunction in vitro [131].

Overall, conflicting results regarding the clinical effectiveness and the loss of protective microbiota species point out that a more defined formula or more stringent diet is needed to effectively use this therapy for adult IBD patients. For pediatric IBD patients, EEN is an established therapy [132]. Even though it has proven effective, understanding the molecular and cellular mechanisms by which EEN promotes its beneficial effects will help to improve the outcome of pediatric IBD by creating optimized formulae.

### Fecal microbiota therapy

The idea of modulating dysbiosis to treat IBD gave rise to the proposal of direct microbiota transplantation with a donor microbiota as a potential therapeutic option (Fig. 1). Fecal microbiota therapy has been successfully used in treating refractory *Clostridium difficile* infection, a colitis triggered by antibiotic-induced dysbiosis [133]. Low frequencies of adverse events have been reported [134]. Fecal microbiota transfer has also shown therapeutic efficacy in inflammatory diseases that are associated with an altered microbiota, such as diabetes and obesity [135].

Data evaluating FMT in IBD still present some limitations. Initial evidence of the role of microbiota transplant in IBD was shown in case reports as having lead to a relief of symptoms in disease refractory to sulfasalazine [136, 137]. In a following retrospective case series, FMT has been shown to induce remission after 4 months of treatment [138]. Further cases have been reported. The proven efficacy of FMT in adults with IBD and a recent phase 1 trial showed a clinical response rate of 67% and clinical remission of 33% of children and young adults after FMT, respectively [139–141]. A more recent study reported the efficacy in CU patients based on intensive dosing of FMT derived from multiple donors [142]. The intense application of 40 FMT during 8 weeks and pooling stool of up to seven donors was the factor which distinguished this trial from recent studies that failed to induce remission, although fecal microbiota changes could be observed [143, 144].

The regimen used by Paramsothy et al. [142] showed a higher microbiota diversity after the fecal transplant, which was also observed 8 weeks after the treatment. However, the invasive and intense form of FMT application is challenging for the patient, and it is still not clear how long patients need to be treated in order to maintain remission.

The role of FMT in the treatment of IBD remains to be fully defined, and more controlled clinical trials are necessary to critically assess its therapeutic effect. It is currently not clear whether antibiotic pretreatment is necessary and which method of administration is the most effective. In the abovementioned study of Pramsothy et al., [142] specific taxa were associated with a positive outcome and others with a negative outcome. These results might be helpful to improve the design of the FMT. Standardized microbiota samples that can be ingested in the form of a pill might display a future option.

## Future perspectives

Taken together, the abovementioned studies point to the fact that the local immune response to intestinal microbiota could be one key mechanism involved in IBD pathogenesis. Numerous risk loci and independent observations described above point to a model where deregulation of the interaction between the immune system and the microbiota favors chronic intestinal inflammation. However, further insights are needed to better decipher the molecular and cellular mechanisms and to develop future therapeutic approaches without the current limitations, such as lack of long-term remission. We hope to have highlighted the need for a unified, scientifically sound basis for future development in this growing field. The identification of food components with an anti-inflammatory function can help to further control IBD and serve as a proof of concept for an approach to induce and maintain remission that could potentially be extended to other immune-mediated diseases. Keeping in mind that each patient’s immune system and microbiota may respond to food components differently, individual dietary approaches will be essential.
